# Excellent oxygen evolution reaction of NiO with a layered nanosphere structure as the cathode of lithium–oxygen batteries†

**DOI:** 10.1039/c7ra12630a

**Published:** 2018-01-16

**Authors:** Hongyu Dong, Panpan Tang, Shiquan Zhang, Xinglu Xiao, Cheng Jin, Yicong Gao, Yanhong Yin, Bing Li, Shuting Yang

**Affiliations:** College of Chemistry and Chemical Engineering, Henan Normal University Xinxiang 453007 China donghy373@163.com; National & Local Engineering Laboratory for Motive Power and Key Materials Xinxiang 453000 China shutingyang@foxmail.com; Collaborative Innovation Center of Henan Province for Motive Power and Key Materials Xinxiang 453000 China; Clean Energy Automotive Engineering Center, Tongji University Shanghai 201804 China lb20016016@163.com

## Abstract

A layered nanosphere structured NiO catalyst was successfully synthesized by a simple and efficient hydrothermal method as a cathode material for lithium–oxygen (Li–O_2_) batteries. Cyclic voltammetry (CV), dual electrode voltammetry (DECV) and chronoamperometry (CA) by rotating ring-disk electrode (RRDE) were carried out to investigate the catalytic activity of this catalyst for the oxygen evolution reaction (OER). The results revealed that the layered nanosphere NiO exhibited excellent electrochemical performance, stability and a typical four-electron reaction as a cathode electrocatalyst for rechargeable nonaqueous Li–O_2_ batteries. The overpotential of the NiO is only up to 0.61 V. X-ray photoelectron spectroscopy (XPS) characterization shows that the Li_2_O_2_ and Li_2_CO_3_ formed during the discharge process and decomposed after charging. Moreover, the cut-off voltage of discharging is about 2.0 V in the NiO-based Li–O_2_ batteries, while the specific capacity is up to 3040 mA h g^−1^. There is no obvious performance decline of the battery after 50 cycles at a current density of 0.1 mA cm^−2^ with a superior limited specific capacity of 800 mA h g^−1^. Herein, the layered nanosphere structured NiO catalyst is considered a promising cathode electrocatalyst for Li–O_2_ batteries.

## Abstract 2

A layered nanosphere structured NiO catalyst was synthesized by a simple and efficient hydrothermal method as a cathode material for lithium–oxygen (Li–O_2_) batteries.

## Graphical abstract

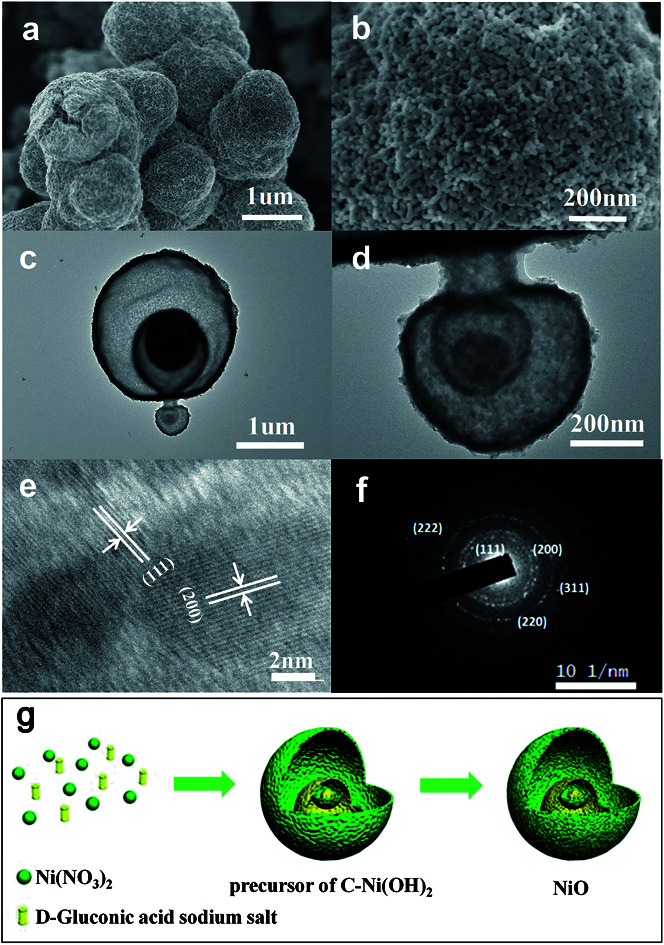

## Introduction

Chemical power sources are attracting more attention in all kinds of renewable energy due to their high conversion rate, ease of building and storage convenience. Lithium–oxygen (Li–O_2_) batteries^1–3^ which possess many advantages, such as a high energy density, low cost and being environmentally benign,^1,4^ are an outstanding way to realize energy goals. Li–O_2_ batteries can store discharge products, such as Li_2_O_2_ and Li_2_CO_3_, in the pores of the cathode and the reactive material, O_2_, in air.^5^ They induce an extremely large theoretical specific energy, varying from 3500 W h kg^−1^ to 5200 W h kg^−1^.^6–8^ In recent years Li–O_2_/air batteries have attracted more and more attention^9–12^ as potential power systems because of their ultrahigh theoretical energy density.^13^ There are multiple scientific challenges which need to be conquered, such as poor capability, low-round-trip efficiency,^1,14^ instability of electrolytes^5,9,14^ and especially the short cycle life.^5,15^ In previous studies,^12,16–18^ it was demonstrated that an exquisite cathode and effective electrocatalyst could accelerate the kinetic reactions and thus improve the overall energy storage efficiency. In particular the slow oxygen reduction reaction (ORR) and oxygen evolution reaction (OER) on the cathode are the centre of these problems. This is directly related to the poor rate capability, large overpotential and electrolyte decomposition.

Discharge processes of Li–O_2_ batteries are roughly classified into three steps. Firstly, oxygen is rapidly dissolved into the electrolyte. Secondly, the dissolved oxygen diffuses to the cathode and is reduced under the action of the catalyst. At the same time the Li metal anode is oxidized. It has been proven that this reaction mechanism can be separated into two steps. The first step is the generation of LiO_2_, which then transforms into Li_2_O_2_. Thirdly, Li_2_O_2_ is deposited on the channel of the cathode.^19–21^ The charging process is the reverse reaction.^16,20,22^ The potential of this whole process needs to be reduced so that charging efficiency can be improved. Therefore, it is very necessary to design an electrocatalyst which is low cost and has high electrical conductivity, stability of structures and high catalytic activity.

Transition metal oxides are composed of single metal oxides and multi-metallic oxides, such as MnO_2_, Co_3_O_4_, MnCo_2_O_4_, CoMn_2_O_4_, Ni-based materials *etc.*^23–29,34^ An alternative metal oxide catalyst should possess some advantages: low price, rich quantity, convenient synthesis and being environmentally benign.^23–25^ NiO in particular exhibits outstanding catalytic activity in Li–O_2_ batteries, as reported recently.^34^ An electrode made of NiO exhibits high thermal stability and electrochemical performance.^30^ There are numerous synthesis methods for layered nanosphere NiO, such as thermal decomposition, chemical precipitation, electrodeposition, sol–gel technology, thermal annealing and cathodic deposition.

In this work, layered nanosphere NiO powder was synthesized by the hydrothermal method which can effectively control the morphology of the product. The prepared layered nanosphere NiO has a hollow structure which can accommodate more discharge products. It also has a larger surface area which can allow more active sites to be established and catalysis reactions to take place effectively. The performance of the superior structure was further investigated in non-aqueous Li–O_2_ batteries.

## Results and discussion

**Fig. 1:**
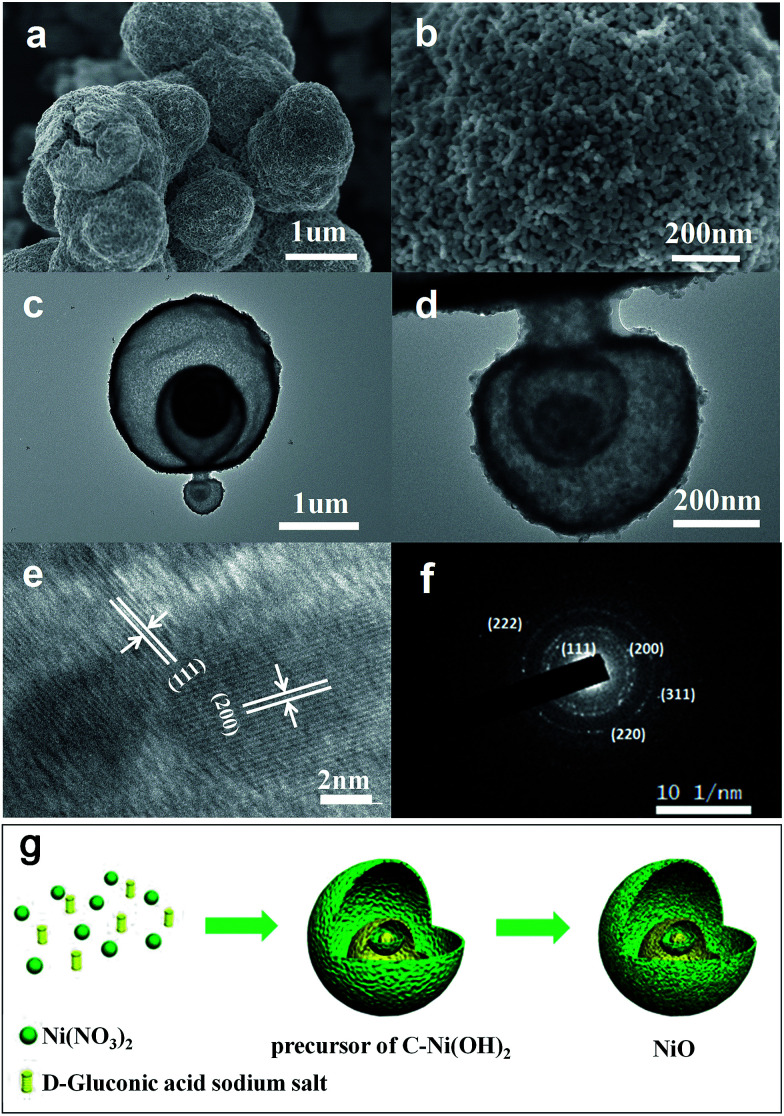
(a) SEM image of NiO. (b) FESEM, (c) TEM and (d) HRTEM images of the layered nanosphere NiO, (e) lattice fringes and (f) hysteretic loop. (g) Schematic illustration of the synthesis process of NiO.

A precursor of C–Ni(OH)_2_ was firstly synthesized using a hydrothermal method. Then the obtained precursor was annealed at 500 °C in air flow to form the NiO with layered nanosphere structure. However, after the annealing treatment, the NiO particles consisted of granules as shown in Fig. 1a and b. This was confirmed by field emission-scanning electron microscopy (FESEM) and transmission electron microscopy (TEM). Fig. 1a shows that the diameter of the nanosphere structured NiO ranges from 2 μm to 3 μm. Fig. 1c is a magnified image of a small area of Fig. 1b which shows that the size of the nanoparticles is 20 nm. The TEM images (Fig. 1c and d) and HRTEM images (Fig. 1e and f) exhibit the stratified structure of the target compound. From Fig. 1c and d it can be seen that the tier thickness of a nanosphere is about 50–100 nm with approximately four layers. FESEM and TEM reveal that the highly porous structure morphology of the layered nanosphere NiO is well preserved after calcination. Selected area electron diffraction (SAED) techniques are performed at several positions on the surface of the nanosphere. Fig. 1e reveals that the crystal lattice *d*-spacing of the layered nanosphere NiO is 2.07 Å, corresponding to the (111) lattice plane. The results display the same diffraction pattern as shown in Fig. 1f, corresponding to the different planes of the NiO crystal particles. Using that, a possible formation mechanism for the layered nanosphere NiO is illustrated in Fig. 1g.

**Fig. 2:**
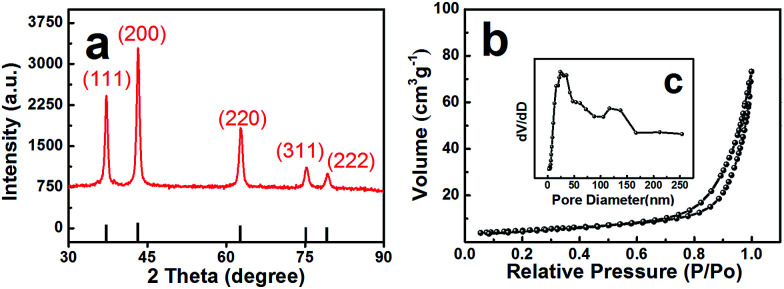
(a) XRD pattern of NiO, (b) the nitrogen adsorption/desorption isotherms and (c) pore-size distribution of NiO.

Fig. 2a shows the X-ray diffractometer (XRD) patterns of the layered nanosphere NiO. The sample was annealed in air at 500 °C for 8 h. All the diffraction peaks of the sample are indexed to the cubic phases of NiO (JCPDS no. 71-1179) such as (111), (200), (220), (311) and (222). No other peaks from impurities are observed. Furthermore, the porous structure was characterized by nitrogen adsorption–desorption measurements. The N_2_ adsorption–desorption isotherm and the corresponding Barrett–Joyner–Halenda (BJH) pore size distribution plots of NiO are observed in Fig. 2b. The adsorption isotherms of NiO are classified as type-V and H3 loop type isotherms. Type-V is obtained with porous adsorbents. The H3 hysteresis loops are attributed to the layer-like particles coming from slit-shaped mesopores. The Brunauer–Emmett–Teller (BET) surface area of the NiO is 26.679 m^2^ g^−1^ which is in accordance with the TEM observations and confirms the mesoporous and macroporous characteristics of the products.

The electrochemical properties, such as the oxygen evolution voltage, have a great effect on the catalytic efficiency of the catalyst. The OER activity of the synthesized NiO was characterized in a three-electrode electrochemical cell with RRDE.

**Fig. 3:**
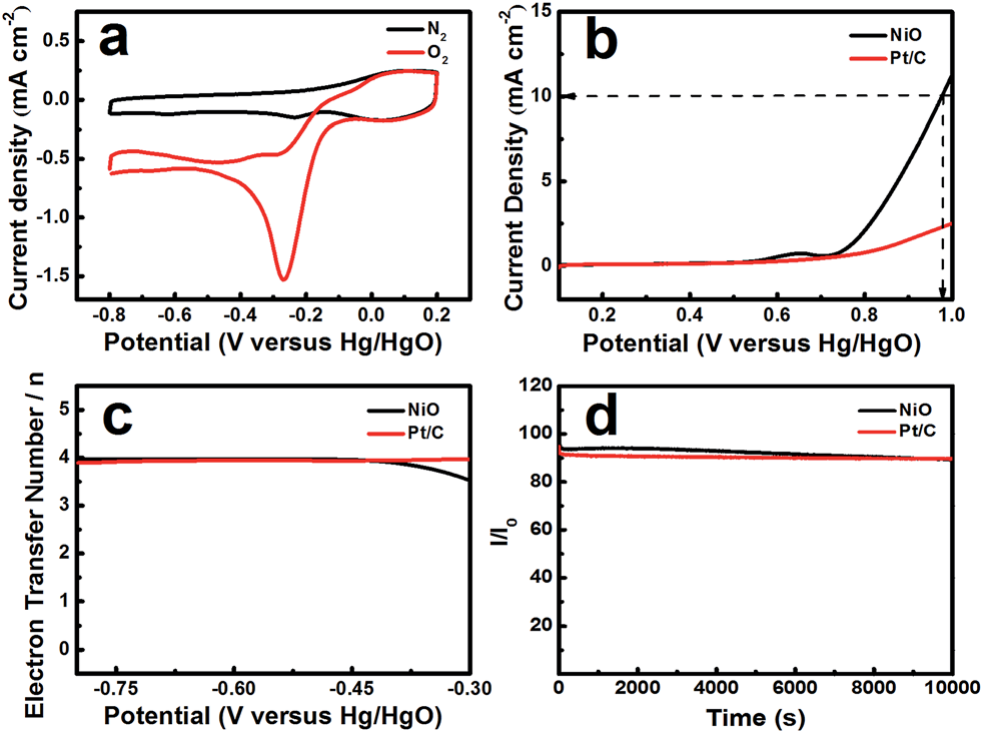
(a) Cyclic voltammograms of NiO (loading ∼0.1 mg cm^−2^) with hydrothermal treatment at 180 °C at a rate of 50 mV s^−1^.(b) Oxygen evolution reaction curves of NiO and Pt/C at a rotation rate of 1600 rpm with a scanning rate of 10 mV s^−1^. (c) The number of electrons transferred during the ORR processes of NiO and Pt/C at a rotation rate of 1600 rpm with a scanning rate of 10 mV s^−1^. (d) Chronoamperometric responses (percentage of current retained *versus* operation time) of NiO and Pt/C on glassy carbon electrodes at a rotation rate of 1600 rpm for 10 000 s in O_2_-saturated 0.1 M KOH.

The electrochemical activity of NiO was tested in an O_2_-saturated 0.1 mol L^−1^ KOH solution. Fig. 3a shows that the CV profiles range from −0.8 to 0.2 V *vs.* Hg/HgO at a scan rate of 10 mV s^−1^. NiO, as the catalyst, displays a reduction peak at −0.27 V. Pt/C, as a comparison, shows a reduction peak at −0.07 V (ESI, Fig. S1†). The peak potential of NiO is negative than that of Pt/C and the current density reaches 1.57 mA cm^−2^, which is lower than that of Pt/C (2.457 mA cm^−2^), but NiO with a layered nanosphere exhibited excellent OER performance. Fig. 3b shows the oxygen evolution reaction curve of NiO. The overpotential (*η*) was defined by the applied potential (*vs.* RHE) minus the water oxidation potential (1.229 V) and the overpotentials that were required to deliver a current density of 10 mA cm^−2^.^31^ When the OER current density reaches 10 mA cm^−2^, the applied potentials reach 0.975 V *vs.* Hg/HgO (*η* = 0.61 V) for the NiO electrode and that of Pt/C is larger than 0.975 V. The OER current densities for NiO and Pt/C reach 11.229 mA cm^−2^ and 2.502 mA cm^−2^ respectively, when the potential is 1 V (*vs.* Hg/HgO). Moreover, the layered nanosphere NiO is better than most of the state-of-the-art nickel-based electrode catalysts in alkaline solutions.^7^ In addition, there are weak anodic waves near 0.643 V (*vs.* Hg/HgO) which correspond to the Ni(OH)_2_/NiOOH redox reaction.^32^

It is demonstrated that the applicable aperture and layered nanosphere NiO structure construct channels for transporting electrons between the conductive substrate and the active surface.^33^ As we know, the OER takes place on the catalyst’s surface and the porosity of the catalyst has a significant effect on the catalytic activity. In the electrochemical reaction, the mesoporous and macroporous structures become more advantageous for the permeation of the electrolyte. Furthermore, the multi-level pore structure becomes more crucial for the gas to escape. During the reaction process, the catalyst easily forms bubbles on the surface which limit the active site. However, the catalyst with a high porosity and layered structure significantly avoids this.^33^ On the whole, a high porosity and layered structure improved the utilization of active sites and sped up the reaction kinetics in NiO. Thus, the layered nanosphere NiO significantly outperforms the Pt/C electrode in the OER.

In order to calculate the electron transfer number of the layered nanosphere NiO, RRDE is employed to get LSV curves at different rotation speeds in O_2_-saturated 0.1 mol L^−1^ KOH at a scan rate of 10 mV s^−1^. A Pt/C catalyst is also tested under the same conditions for comparison. As shown in Fig S2 and S3,† the curves at the current density near 0 mA cm^−2^ are coursed by ring-current. The limiting current density of NiO and Pt/C increases with the increase of rotation speed. At 1600 rpm, the limiting current density of NiO reaches 2.51 mA cm^−2^ and is negative than that of Pt/C (5.43 mA cm^−2^).

The electron transfer number was calculated from the rotating ring-disk electrode (RRDE) date using the following formula (1):1
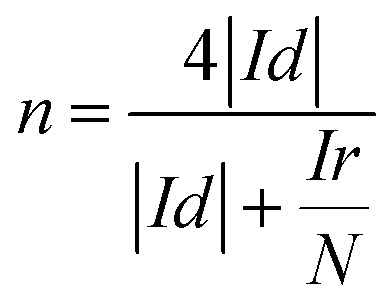


As can be seen from Fig. 3c the electron transfer number is about 3.92, which is slightly lower than that of Pt/C in the potential range from −0.8 V to −0.3 V, indicative of a four-electron main process. It can be seen in Fig. 3d that the layered nanosphere NiO exhibits better stability than Pt/C, this is related to the specific structure of NiO. This also demonstrates that the layered nanosphere NiO maintains a stable structure after 10 000 seconds.

**Fig. 4:**
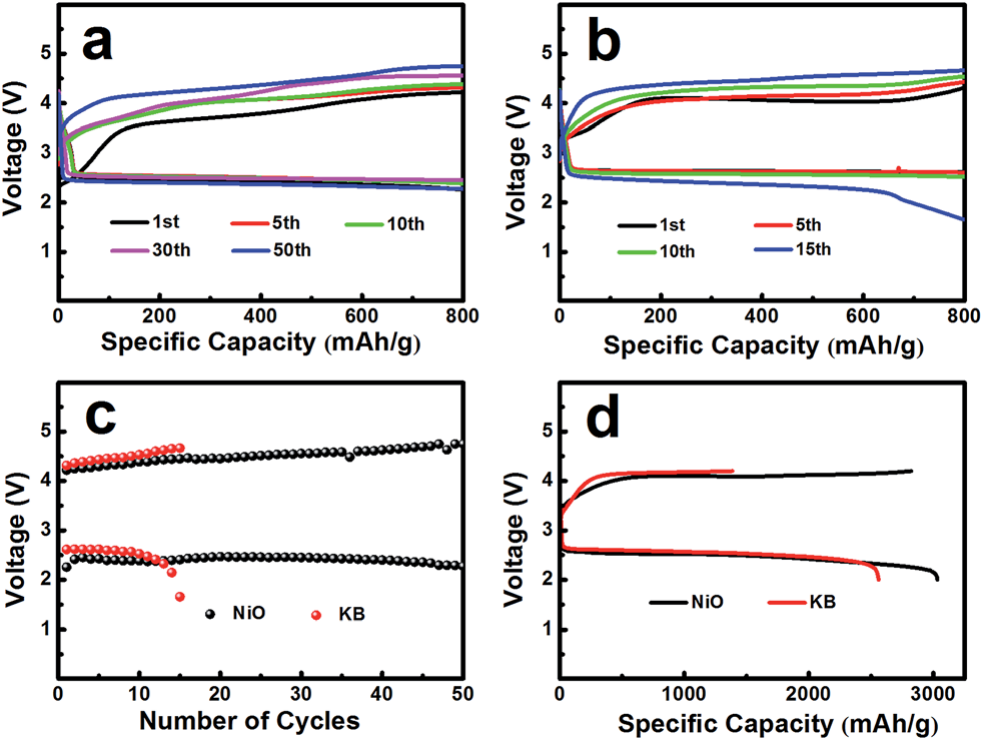
(a) NiO treated hydrothermally at 180 °C and (b) KB as a cathode catalyst at 0.1 mA cm^−2^ in the voltage range of 2.0–4.2 V *vs.* Li^+^/Li with a limited specific capacity of 800 mA h g^−1^ . (c) NiO and KB as the cathode catalyst with the cycle number of the Li–O_2_ batteries corresponding to NiO and KB. (d) Galvanostatic discharging/charging curves of Li–O_2_ batteries with NiO and KB as the cathode catalyst at a current density of 0.1 mA cm^−2^ in the voltage range of 2.0–4.2 V *vs.* Li^+^/Li.

The electrochemical performances of the NiO cathode for Li–O_2_ batteries were assessed in a pressure-tight test chamber which was filled with oxygen to 99.99%. Fig. 4a and b show the cycling performance of the NiO cathode, which was tested at a current density of 0.1 mA cm^−2^ under a limited specific capacity of 800 mA h g^−1^ . The NiO electrode was discharged and charged for at least 50 cycles above 2.0 V. In contrast, the Ketjenblack (KB) electrode only lasted for 15 cycles with 0.1 mA cm^−2^ under a limited specific capacity of 800 mA h g^−1^ which is obviously less than that of the NiO electrode. This demonstrates that the NiO electrode delivers a better cycling performance than KB at a lower charge potential, and that the durability of the NiO catalyst is far better than that of KB, providing a relatively better capacity retention. NiO is responsible for the bifunctional catalytic activities of the cathode. The layered nanosphere NiO not only assists the flow of oxygen and the infiltration of the electrolyte, but it also prevents channel clogging and structure collapse in the discharge products. The cut-off voltages of NiO and KB are presented in Fig. 4c which correspond to Fig. 4a and b. The batteries are set for galvanization cycling with a current density of 0.1 mA cm^−2^. Electrochemical tests of the specific capacity are achieved in the voltage range from 2.0 to 4.2 V. Fig. 4d presents the maximal specific capacities of NiO and KB, which are acquired during the first discharging and charging process. The NiO cathode attains a discharge specific capacity of 3040 mA h g^−1^, revealing a better initial capacity than the KB electrode. Generally, transition metal oxides, such as NiO, exhibit poor electrical conductivity however KB, working as a favorable carbon material, exhibits fine electrical conductivity. In spite of this, the NiO also exhibits a lower potential than KB, and the first cycle efficiency reached 98.3%, more than the 54.8% of KB. The outstanding performance of NiO is primarily due to the layered nanosphere structure and excellent OER activity.

**Fig. 5:**
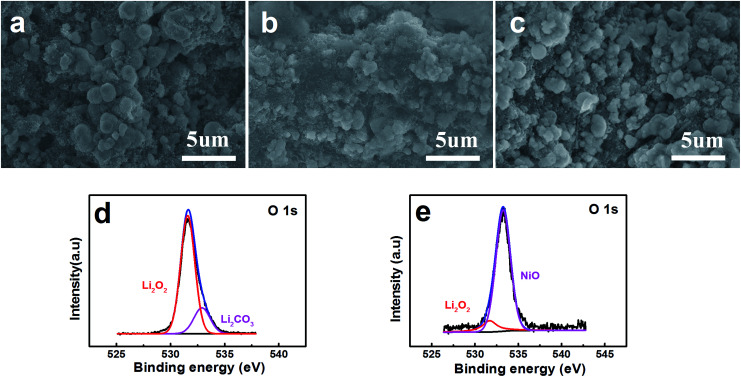
FESEM images of (a) a pristine NiO electrode, (b) a discharged NiO electrode and (c) a charged NiO electrode at 0.1 mA cm^−2^ in the voltage range of 2.0–4.2 V *vs.* Li^+^/Li with a limited specific capacity of 800 mA h g^−1^ . (d) and (e) XPS spectra corresponding to the discharged NiO electrode and charged NiO electrode respectively.

FESEM images show the surface of the pristine layered nanosphere NiO cathode in Fig. 5a. The layered nanosphere NiO can be seen obviously and it is clearly observed that the reaction compounds deposit homogenously on the surface of the layered nanosphere NiO after discharging with a limited specific capacity of 800 mA h g^−1^.^34^ However, it is evidently discovered that the reaction compounds are almost completely faded out in Fig. 5c. This demonstrates that the NiO cathode has a distinguished ability to promote the decomposing of the reaction compounds. From the viewpoint of the reaction, NiO has exhibited excellent performance of the OER. Meanwhile, in order to probe the function of the NiO, the discharging products (discharged to 800 mA h g^−1^ at a current density of 0.1 mA cm^−2^) are characterized and analyzed by X-ray photoelectron spectroscopy (XPS). As observed in Fig. 5d and e, the compounds Li_2_O_2_ and Li_2_CO_3_ are detected on the composite NiO cathode. After charging, a great amount of Li_2_O_2_ has disappeared and the NiO is exposed to the surface of the cathode (Fig. 5e). This reveals that the reaction compound in Fig. 5b is Li_2_O_2_ and it can be decomposed entirely on the surface of the NiO cathode, implying that NiO promotes the decomposition of Li_2_O_2_.

**Fig. 6:**
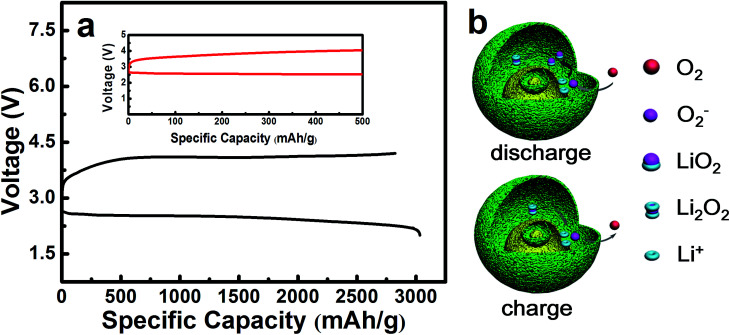
(a) Galvanostatic discharge/charge curves of Li–O_2_ batteries with a NiO catalyst at a current density of 0.1 mA cm^−2^ in the voltage range of 2.0–4.2 V *vs.* Li^+^/Li. (b) Illustration of the catalytic reaction mechanism for the layered nanosphere NiO in Li–O_2_ batteries.

The maximal specific capacity of NiO is 3040 mA h g^−1^ which is acquired during the first discharging and charging process, as shown in Fig. 6a. The reaction mechanism is illustrated in Fig. 6b, corresponding to eqn (2)–(6) as follows. This explains why the layered nanosphere NiO with more pore channels can effectively store the discharging products and keep the oxygen channel unobstructed. It has been found by investigation that transition metal oxides with porous structure display outstanding OER performances, owing to particular pathways^35–38^ in accordance with the results of our studies.

There are three probable discharge reactions in the Li–O_2_ batteries and eqn (2) reveals the discharging process which has been studied. It concludes that the oxygen reduction product in Li–O_2_ batteries is LiO_2_. Then, the LiO_2_ chemically decomposes into Li_2_O_2_ and O_2_, as shown in eqn (3), which raises the nonreversible electrochemical process of the ORR.^35,39^

2 Li^+^ + O_2_ + e^−^ = LiO_2_ (2.5 V)

3 2LiO_2_ = Li_2_O_2_ + O_2_

The charging process is shown in eqn (4)–(6). It may contain one or multifarious steps as mentioned in the literature.^40,41^

4 Li_2_O_2_ (core) = 2Li^+^ + O_2_ + 2e^−^ (3.8–4.1 V)

5 O_2_^−^ = O_2_ + e^−^ (3.2–3.6 V)

6 Li_2_O_2_ (shell) = 2Li^+^ + O_2_ + 2e^−^ (3.2–3.6 V)

In Fig. 6a, it is precisely observed that Li_2_O_2_ decomposes at 3.2–3.6 V in accordance with eqn (6). This probably corresponds to the charging specific capacity of 500 mA h g^−1^. The next reaction could take place in the core of the layered nanosphere NiO, relating to eqn (4). As a result, the charging specific capacity increases from 500 mA h g^−1^ to 3040 mA h g^−1^, as shown in Fig. 6a. It is inferred that NiO effectively promotes the decomposition of Li_2_O_2_ on the shell of the layered nanosphere NiO electrocatalyst as there are so many channels in which electrochemical reaction can occur in the layered nanosphere structure. The catalytic reaction mechanism of the layered nanosphere NiO electrocatalyst is illustrated in Fig. 6b. The utilization of active sites is enhanced by the high porosity and layer structure and consequently the reaction kinetics are improved. Moreover, the excellent OER activity of the layered nanosphere NiO as a cathode catalyst is better than that of other NiO-based materials.^30^ This demonstrates that the special structure of the layered nanosphere NiO shows preeminent OER performance.

## Conclusions

A layered nanosphere NiO was successfully synthesized by a facile hydrothermal method and heated appropriately. The hollow layered nanosphere NiO exhibited excellent performance in terms of OER activity and superior stability in contrast to commercial Pt/C. The NiO cathode attained a discharging specific capacity of 3040 mA h g^−1^, revealing a better initial specific capacity than KB. Li–O_2_ batteries made with the NiO electrocatalyst ran for 50 cycles and were very stable compared to those made with the KB electrode. There are multiple step reactions in the discharging process at different voltages which demonstrates that there are many channels in the layered nanosphere NiO. The special structure prospectively results in a rapid and effective electrocatalytic reaction. The layered nanosphere NiO is a promising electrocatalyst for Li–O_2_ batteries.

## Experimental

### Materials

NiO was synthesized by a hydrothermal method where d-gluconic acid sodium salt (Aladdin) and Ni(NO_3_)_2_·6H_2_O (Sinopharm Chemical Reagent Co., Ltd) (with a molar ratio of 1 : 1) were mixed in deionized water under magnetic stirring for 1 h to form a transparent solution. Then, the final mixture was transferred to a high pressure autoclave at 180 °C for 24 h and hydrothermally treated to form a precursor of C–Ni(OH)_2_. After that the C–Ni(OH)_2_ precursor was carbonized at 500 °C for 8 h with a heating rate of 5 °C min^−1^.

### Electrochemical measurements

The catalyst slurry was made with 5 mg NiO or Pt/C, 5 mg acetylene black (AB), 35 μL Nafion and 95 uL 99.9% ethyl alcohols. The catalyst slurry was dropped onto a glassy carbon rotating disk electrode of 7 μL, after being under ultrasonic conditions for half an hour. The OER performance was investigated to measure the electrocatalytic activities of NiO using a three-electrode test system with a platinum-wire electrode as the counter electrode and a Hg/HgO electrode as the reference electrode. The Hg/HgO electrode was adjusted to a reversible hydrogen electrode (RHE). All electrodes were immersed in a 0.1 M KOH solution at room temperature.

### Li–O_2_ battery measurements

The electrochemical performances of the materials were evaluated *via* CR2032 coin batteries. A mixture of 80 wt% active materials and 10 wt% conductive agents (Super P) with 10 wt% polyvinylidene fluoride (PVDF) in *N*-methyl-2-pyrrolidone solvent was ground into a slurry. Then the slurry was pasted onto carbon paper under vacuum at 60 °C overnight. Dried pre-cathodes were cut to 12 mm in diameter (the mass of the active material is around 0.8–1.2 mg). CR2032 coin batteries were assembled in a glovebox with argon gas. Glass fiber is used as the separator and lithium foil as the anode. There are several holes in the shell for oxygen flow. An electrolyte was compounded with 1 M lithium LiCF_3_SO_3_/TEGDME. The batteries were put into an oxygen-filled test chamber (1 atm) for 1 h at room temperature.

### Characterization

The surface features of the samples were detected by field emission-scanning electron microscopy (FESEM, SU-8010, HITACHI). In order to precisely measure the thickness of the nanosphere layer, transmission electron microscopy (TEM, JEM-2100, JEOL) was performed, operating at 200 kV with selected area electron diffraction (SAED). The crystal structure of the NiO with multi-walled hollow spheres was surveyed by an X-ray diffractometer (Bruker D2 PHASER) with Cu Kα (*λ* = 1.541 Å) radiation. The surface area of NiO was tested by the Brunauer–Emmett–Teller (BET) method with N_2_ adsorption–desorption isotherms. The porosity of NiO was calculated by the Barrett–Joyner–Halenda (BJH) method based on N_2_ adsorption–desorption isotherms (nitrogen adsorption–desorption isotherms were measured at 77 K with a TristartII3020 Multichannel automatic specific surface area analyzer). The elemental compositions of the discharge products were characterized by X-ray photoelectron spectroscopy (XPS) using an ESCALAB 250 spectrometer equipped with Al Kα radiation (300.0 eV).

## Conflicts of interest

There are no conflicts to declare.

## Supplementary Material

RA-008-C7RA12630A-s001
