# Supplementary material for: Excellent oxygen evolution reaction of NiO with a layered nanosphere structure as the cathode of lithium–oxygen batteries
Source: RSC Adv. 2018 Jan 16;8(7):3357–63. doi: 10.1039/c7ra12630a (PMC9077675; doi:10.1039/c7ra12630a)
Supplement: RA-008-C7RA12630A-s001 [file RA-008-C7RA12630A-s001.pdf]

Electronic Supplementary Material (ESI) for RSC Advances.
This journal is © The Royal Society of Chemistry 2018

Supporting informantion

Excellent Oxygen Evolution Reaction of NiO with Layered Nanosphere as the Lithium-Oxygen Batteries Cathode

Hongyu Dong,*[abc] Panpan Tang,[abc] Shiquan Zhang,[abc] Xinglu Xiao,[abc] Cheng Jin,[abc] Yicong Gao,[abc] Yanhong Yin,[abc] Bing Li*[d] and Shuting Yang*[abc]

[a] College of Chemistry and Chemical Engineering, Henan Normal University, Xinxiang 453007, China

[b] National & Local Engineering Laboratory for Motive Power and Key Materials, Xinxiang 453000, China

[c] Collaborative Innovation Center of Henan Province for Motive Power and Key Materials, Xinxiang 453000, China

[d] Clean Energy Automotive Engineering Center, Tongji University，Shanghai 201804 China

*Corresponding Author. Tel.: (+86)-373-3326439

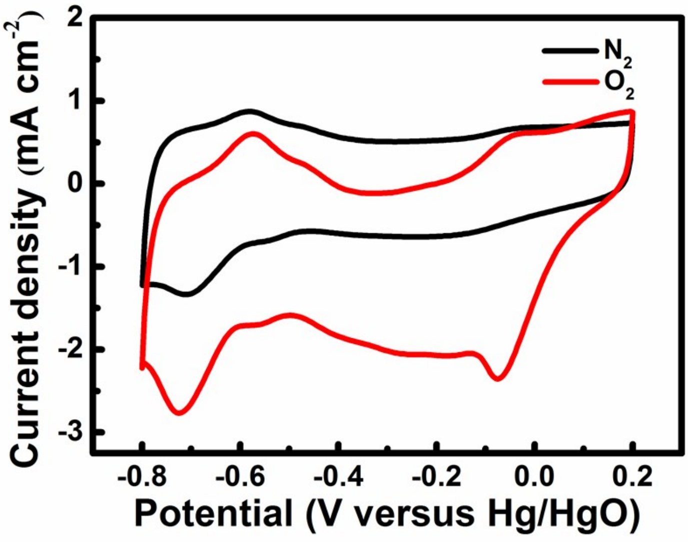

Figure S1 Cyclic voltammograms of Pt/C (loading ~0.1 mg cm^{-2}) at a rate of 50 mV s^{-1}.

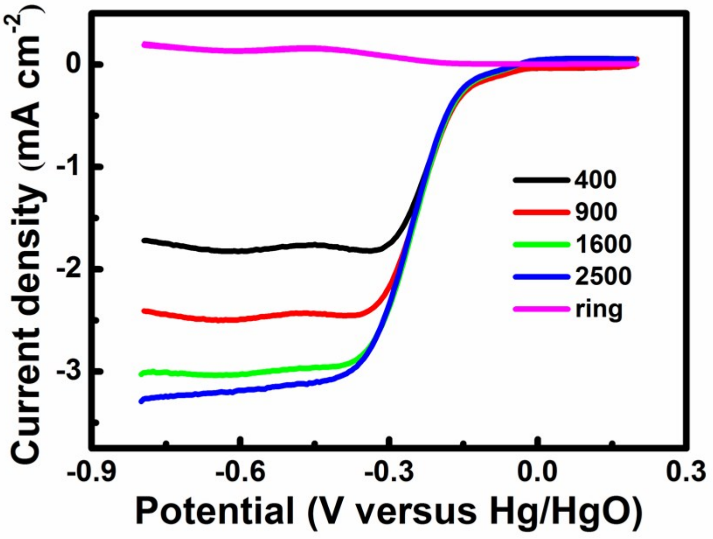

Figure S2 Oxygen reduction reaction curves of NiO with RRDE at a rotation rate of 400 rpm, 900 rpm, 1600 rpm and 2500 rpm with scanning rate of mV s^{-1}.

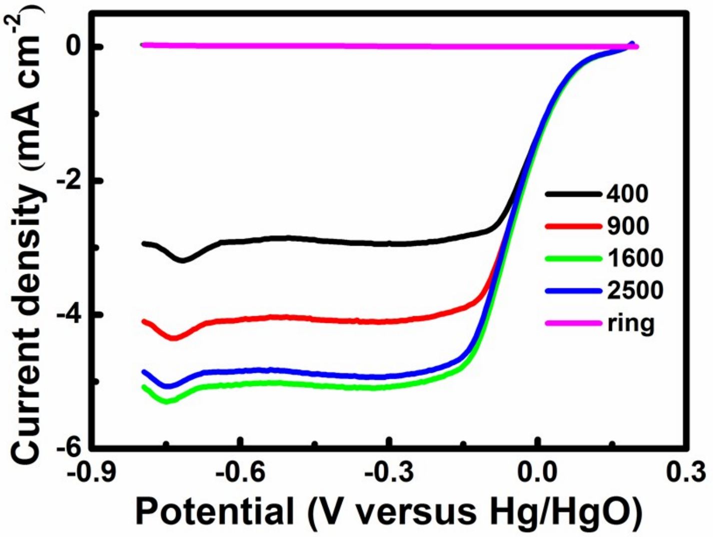

Figure S3 Oxygen reduction reaction curves of Pt/C with RRDE at a rotation rate of 400 rpm, 900 rpm, 1600 rpm and 2500 rpm with scanning rate of 10 mV s^{-1}.
